# Associations of cholecystectomy with metabolic health changes and incident cardiovascular disease: a retrospective cohort study

**DOI:** 10.1038/s41598-024-53161-6

**Published:** 2024-02-08

**Authors:** Sangwoo Park, Seogsong Jeong, Sun Jae Park, Jihun Song, Sung Min Kim, Jooyoung Chang, Seulggie Choi, Yoosun Cho, Yun Hwan Oh, Ji Soo Kim, Young Jun Park, Joung Sik Son, Joseph C. Ahn, Sang Min Park

**Affiliations:** 1 Department of Biomedical Sciences, Seoul National University Graduate School, Seoul, South Korea; 2 Department of Biomedical Informatics, CHA University School of Medicine, Seongnam, South Korea; 3 Department of Family Medicine and Biomedical Sciences, College of Medicine, Seoul National University, 101 Daehak-ro, Jongno-gu, Seoul, South Korea; 4 Department of Internal Medicine, Seoul National University Hospital, Seoul, South Korea; 5 Total Healthcare Center, Kangbuk Samsung Hospital, School of Medicine, Sungkyunkwan University, Seoul, Republic of Korea; 6 Department of Family Medicine, Chung-Ang University Gwangmyeong Hospital, Chung-Ang University College of Medicine, Gwangmyeong-si, South Korea; 7 International Healthcare Center, Seoul National University Hospital, Seoul, Republic of Korea; 8 Medical Research Center, Genomic Medicine Institute, Seoul National University College of Medicine, Seoul, South Korea; 9 Department of Family Medicine, Korea University Guro Hospital, Seoul, Republic of Korea; 10 Division of Gastroenterology and Hepatology, Mayo Clinic, Rochester, MN USA

**Keywords:** Biomarkers, Cardiology, Diseases, Health care, Medical research

## Abstract

Although some studies conducted about the risk of cholecystectomy and cardiovascular disease, there was a limit to explaining the relationship. We investigated the short-term and long-term relationship between cholecystectomy and cardiovascular disease, and evidence using the elements of the metabolic index as an intermediate step. It was a retrospective cohort study and we used the National Health Insurance Service database of South Korea between 2002 and 2015. Finally, 5,210 patients who underwent cholecystectomy and 49,457 at 1:10 age and gender-matched controls of subjects were collected. The main results was estimated by Multivariate Cox proportional hazard regression to calculate the hazard ratio (HR) with 95% confidence interval (CI) for risk of cardiovascular disease after cholecystectomy. Regarding short-term effects of cholecystectomy, increased risk of cardiovascular disease (aHR 1.35, 95% CI 1.15–1.58) and coronary heart disease (aHR 1.77, 95% CI 1.44–2.16) were similarly seen within 2 years of surgery. When analyzing the change in metabolic risk factors, cholecystectomy was associated with a change in systolic blood pressure (adjusted mean [aMean]: 1.51, 95% CI: [− 1.50 to − 4.51]), total cholesterol (aMean − 14.14, [− 20.33 to 7.95]) and body mass index (aMean − 0.13, [− 0.37 to 0.11]). Cholecystectomy patients had elevated risk of cardiovascular disease in the short-term, possibly due to the characteristics of the patient before surgery. The association of cholecystectomy and cardiovascular disease has decreased after 2 years in patients who underwent cholecystectomy, suggesting that because of improvement of metabolic health, cholecystectomy-associated elevation of cardiovascular disease risk may be ameliorated 2 years after cholecystectomy.

## Introduction

Cholecystectomy is one of the most common organ removal surgery and is performed for a variety of indications including symptomatic gallstones, acute and chronic cholecystitis, and gallbladder polyps^1^. While cholecystectomy is typically viewed as a simple and benign operation with no major long-term effects on the patients’ health, it is in fact associated with significant physiologic changes in digestive metabolism. The gallbladder plays an important role in the storage and release of bile for emulsification and absorption of dietary fat^2^. The integrity of enterohepatic circulation is critical for maintaining homeostasis of the digestive tract and balance of the intestinal microbiota. Surgical removal of the gallbladder has been shown to profoundly alter the enterohepatic circulation and the type and number of intestinal microbiota^3,4^. Therefore, these changes in the body can affect lipid metabolism^2,5^.

In 2020, cholecystectomy was the fifth most common surgery in South Korea with over 84,000 cases which has increased from the previous year. Despite of the rising trend, there remains a lack of understanding on the mechanism of either short- or long-term effects of cholecystectomy on the risk of systemic diseases. Previous papers showed that the risk of colon cancer increased after cholecystectomy, especially in women^6^. In addition, a study of metabolic syndrome with non-alcoholic fatty liver indicated that patients with cholecystectomy had an increased risk of metabolic syndrome and they also had an higher risk of non-alcoholic fatty liver disease^7^. However, these studies were unable to explain the intermediate mechanism associated with the disease after cholecystectomy^2,5,8–15^.

Cardiovascular disease is among the leading causes of death globally^16^. Preventative efforts focus on addressing and modifying metabolic syndrome, which is the biggest risk factor for cardiovascular disease^17,18^. It is plausible that post-surgical changes in metabolism may influence patients’ risk of cardiovascular disease^17^. We hypothesized that physiologic changes that take place after cholecystectomy may affect the indicators of metabolic syndrome and consequently the risk of cardiovascular disease. The aim of this study was to analyze the short-term changes in the indicators of metabolic syndrome and the short- and long-term risk of cardiovascular disease following cholecystectomy.

## Methods

### Data source

This study was a retrospective cohort study. It conducted by using the database of National Health Insurance Service (NHIS-2022-2-088) in South Korea between January 1st, 2002 and December 31st, 2015^19^. As South Korea has a universal healthcare system, most Koreans have health insurance and are represented in the NHIS database. The NHIS database contains information on patients’ gender, age, insurance grade due to income, residence, year of examination, medical practice experienced, prescription, dental treatment, oriental medicine treatment, and the name of the disease diagnosed with ICD-10 code (the 10th revision of the International Statistical Classification of Diseases). The NHIS conducts health checkups once every two years for all subjects over the age of 40. These follow-up data can determine changes in smoking status, changes in drinking volume, exercise amount per week, blood pressure, body mass index, total cholesterol, etc.

### Study population and design

**Figure 1:**
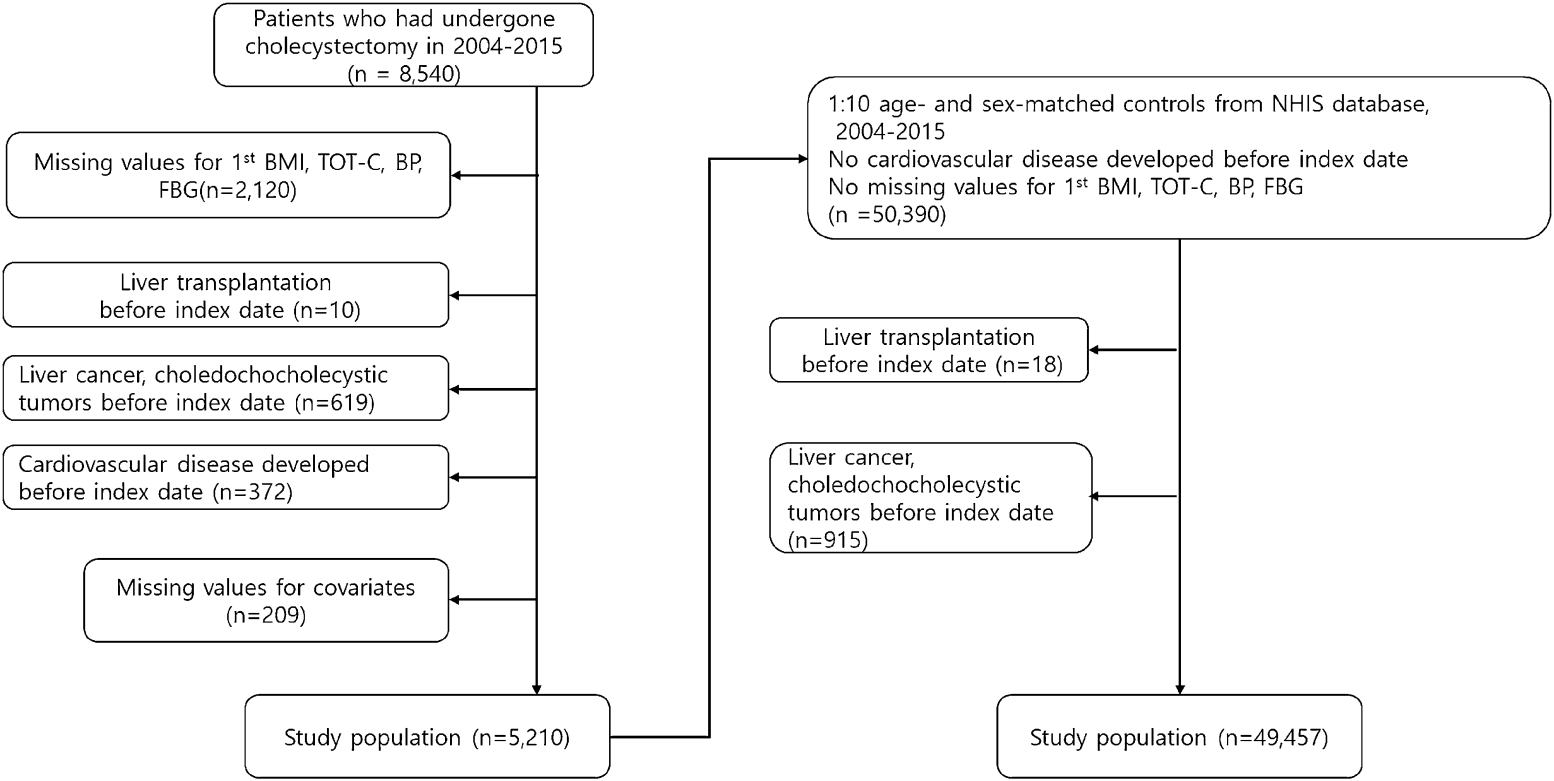
Title flow diagram of the study subjects.

The National Health Insurance Service's health examination database spans from years 2002 to 2015, but we extracted patients who underwent cholecystectomy between years 2004 and 2013 because we needed examination data within 2 years before and after cholecystectomy. In Fig. 1, we first identified 8540 patients who underwent cholecystectomy between January 1st, 2004 and December 31st, 2015. We excluded 2120 patients whose measurements on systolic blood pressure, diastolic blood pressure, total cholesterol and body mass index were not available, and excluded 209 patients whose gender and age information were not available. In addition, we excluded 10 patients with history of liver transplantation before the surgery date, 619 patients diagnosed with hepatobiliary malignancies during the study period, and 372 patients diagnosed with cardiovascular disease before cholecystectomy, leaving us with a total case population of 5210 patients. The cholecystectomy group was then matched on age and gender to a control group of 50,390 patients using a 1:10 matching ratio. Using the same exclusion criteria, the final number of patients in the control group was reduced to 49,457. .

We defined cholecystectomy as patients with surgical records with surgical procedure code Q7380. The population group was extracted again to see changes in health within 2 years immediately after cholecystectomy and within the previous 2 years. At this time, within 2 years immediately after cholecystectomy, the subjects did not die, and subjects in a slightly healthy state were extracted because subjects without cardiovascular disease were selected. At this time, a total of 4007 people were extracted, excluding 1204 subjects without secondary measurements among previously extracted case population of 5210 patients. For this group, 40,070 was extracted by matching 1:10 by age and gender for subjects with both primary and secondary examinations, no experience of cardiovascular disease, gallbladder cancer, biliary cancer, and liver cancer, and no missing values of covariates.

### Key variables

 The primary exposure variable was cholecystectomy as defined by the surgical code Q7380. The dependent variables included fasting blood sugar, blood pressure and total cholesterol and body mass index. Covariates included gender, age, income, number of medium-intensity exercise, smoking, drinking amount, and Charlson Comorbidity Index (CCI). The number of medium-intensity exercise refers to the sum of the number of intense exercise times within 1 week for more than 20 min and the number of exercise times within 1 week for more than 30 min. Cardiovascular disease includes coronary heart disease, total stroke. The primary outcome of this study was the incidence of cardiovascular disease (CHD and TS)^20–23^. Patients with cardiovascular disease were defined as those who were hospitalized for more than 2 days due to cardiovascular disease during the follow-up period from immediately after cholecystectomy to December 31st, 2015 or whose cause of death was cardiovascular disease^24–27^.

### Statistical analysis

Participants were analyzed by the Cox proportional hazards regression models that were adjusted for the following potential confounding factors^28^. Hazard ratios calculated by Cox proportional hazards regression analysis. The results was calculated after adjustments for sex, age, income, smoking status, blood pressure, alcohol habits, fasting serum glucose, body mass index (BMI), total cholesterol, Charlson comorbidity index. Next, multiple regression analysis to mean difference which is the variance of metabolic health was performed with age and gender as correction variables. Finally, multiple regression analysis was calculated with age, sex, smoking status, income, alcohol consumption, medium-intensity exercise count, and comorbid disease index as correction variables^2,29–31^. The incidence of cardiovascular disease between the two groups was calculated during person-years of follow-up, and the results were used to estimate the hazard ratio (HR) and 95% confidence interval (CI)^32^. When the P value was less than 0.05, the association was considered statistically significant. All missing values were excluded from the analysis, and SAS Enterprise Guide version 7.1 was used for the analysis.

### IRB approval

The Institutional Review Board of Seoul National University Hospital approved this study before researching (IRB number: E-2108-136-1246). This study was conducted to the extent that it met the ethical standards of the Helsinki Declaration, which was declared in 1964, and revised thereafter, with the informed consent of all participants. This database was anonymized, prior to distribution by the NHIS, by strict confidentiality guidelines.

## Results

### Baseline characteristics

Table 1 shows the baseline characteristics of two groups in this study. This cohort included 5210 patients who underwent cholecystectomy and 49,457 age-and gender-matched controls without cholecystectomy. Both groups had an average age of 59 years and had the same male-to-female ratio of 57% to 43%.

**Table 1:** Descriptive statistics of the participants in the National Health Insurance Service.

	No cholecystectomy (n = 49,457)	Cholecystectomy (n = 5210)	*p* value
Age, years	59 (53–68)	59 (53–68)	0.046
Sex, n (%)			0.920
Men	28,167 (57.0)	2971 (57.0)	
Women	21,290 (43.1)	2239 (43.0)	
Household income^a^, n (%)			< 0.0001
First (highest)	15,116 (30.6)	1995 (38.3)	
Second	12,709 (25.7)	1483 (28.5)	
Third	11,468 (92.0)	1003 (19.3)	
Fourth (lowest)	10,164 (20.6)	729 (14.0)	
Body mass index, kg/m^2^	23.8 (21.9–25.7)	24.3 (22.5–26.2)	< 0.0001
Systolic blood pressure, mmHg	126 (117–136)	127 (117–137)	0.051
Diastolic blood pressure, mmHg	80 (70–84)	80 (70–85)	0.036
Total cholesterol, mg/dL	197 (174–222)	195 (171–221)	0.001
Fasting serum glucose, mg/dL	95 (87–106)	96 (87–108)	< 0.0001
Cigarette smoking, n (%)			0.343
Never smoker	33,174 (67.1)	3444 (66.1)	
Past smoker	7462 (15.9)	817 (15.7)	
Current smoker	8821 (17.8)	949 (18.2)	
Alcohol consumption, n (%)			< 0.0001
None	33,116 (67.0)	3640 (69.9)	
1–2 times/week	10,385 (21.0)	925 (17.8)	
3–4 times/week	3771 (7.6)	380 (7.3)	
≥ 5 times/week	2185 (4.4)	265 (5.1)	
Physical activity, n (%)			0.349
None	24,482 (49.5)	2608 (50.1)	
1–2 times/week	10,080 (20.4)	1091 (20.9)	
3–4 times/week	6431 (13.0)	664 (9.4)	
≥ 5 times/week	8464 (17.1)	847 (16.3)	
Charlson comorbidity index, n (%)			< 0.0001
0	19,427 (39.3)	1226 (23.5)	
1	15,788 (31.9)	1609 (30.9)	
≥ 2	14,242 (28.8)	2375 (45.6)	

Data are presented as median (interquartile range) unless otherwise specified.

^a^Proxy for socioeconomic status based on the insurance premium of the National Health Insurance Service.

Median value of BMI showed 24.3 for the group who experienced cholecystectomy and 23.8 for the unexperienced group, and these values were the primary examination data before cholecystectomy, so the difference in BMI was statistically significant before surgery and the group requiring cholecystectomy tended to be a little higher. Fasting serum glucose showed a higher tendency in patients with cholecystectomy. The proportion of the smoking population was classified into the population who never smoked, the population who smoked before but now, and the population who continued to smoke since before. The percentage of drinking and exercise was determined by the number of times per week, and the Charlson comorbidity index refers to the prevalence of other diseases (Supplementary Table 1).

### Cholecystectomy and cardiovascular outcomes

In Table 2, multivariable Cox proportional hazards regression, patients who underwent cholecystectomy had significantly elevated overall risk of CHD (aHR 1.22, 95% CI 1.07-1.40). When focusing on short-term cardiovascular risk within 2 years of cholecystectomy, cholecystectomy was associated with increased risk in both CVD (aHR 1.35, 95% CI 1.15-1.58) and CHD (aHR 1.77, 95% CI 1.44-2.16). However, there were no statistically significant associations between cholecystectomy and long-term risk of CVD or CHD greater than 2 years after the surgery^10^.

**Table 2:** HR of short-term and long-term risk of cardiovascular disease by cholecystectomy.

	No cholecystectomy	Cholecystectomy	*p* value
No. of participants, n	49,457	5210	
Cardiovascular disease			
Events, n	3487	460	
Person-years	215,486	22,169	
aHR (95% CI)^a^	1.00 (reference)	1.08 (0.98–1.19)	0.131
Coronary heart disease			
Events, n	1599	252	
Person-years	218,766	22,556	
aHR (95% CI)^a^	**1.00 (reference)**	**1.22 (1.07–1.40)**	**0.004**
Total stroke			
Events, n	2087	234	
Person-years	218,506	22,827	
aHR (95% CI)^a^	1.00 (reference)	0.93 (0.81–1.06)	0.270
Short-term (< 2 years) risk			
Cardiovascular disease			
Events, n	1170	190	
Person-years	85,844	8865	
aHR (95% CI)^a^	**1.00 (reference)**	**1.35 (1.15–1.58)**	**0.001**
Coronary heart disease			
Events, n	542	121	
Person-years	86,251	8917	
aHR (95% CI)^a^	**1.00 (reference)**	**1.77 (1.44–2.16)**	**< 0.0001**
Total stroke			
Events, n	665	76	
Person-years	86,213	8988	
aHR (95% CI)^a^	1.00 (reference)	0.98 (0.77–1.25)	0.860
Long-term (≥ 2 years) risk			
Cardiovascular disease			
Events, n	2317	270	
Person-years	214,343	22,027	
aHR (95% CI)^a^	1.00 (reference)	0.95 (0.83–1.08)	0.434
Coronary heart disease			
Events, n	1057	131	
Person-years	218,245	22,472	
aHR (95% CI)^a^	1.00 (reference)	0.95 (0.78–1.14)	0.557
Total stroke			
Events, n	1422	158	
Person-years	217,842	22,763	
aHR (95% CI)^a^	1.00 (reference)	0.91 (0.77–1.07)	0.251

^a^Hazard ratios calculated by Cox proportional hazards regression analysis after adjustments for age, household income, alcohol consumption, body mass index, systolic blood pressure, fasting serum glucose, total cholesterol, Charlson comorbidity index.

In the long-term analysis, cholecystectomy patients represented an otherwise healthy sub-population with at least 2-years of survivorship, which may introduce type II error. To minimize survivor bias, however, both the case and controls were given at least 2-years of survival time.

*aHR* adjusted hazard ratio, *CI* confidence interval.

Significant values are in bold.

We have performed several sensitivity analyses for better understanding of the association between cholecystectomy and risk of cardiovascular disease In Table 3, in Table 4, cholecystectomy was not associated with statistically significant differences in short-term or long-term risk of CVD among women, while it was associated with significantly increased short-term risk of CVD among men (aHR 1.39, 95% CI 1.15–1.69). Finally, when stratified by body mass index, cholecystectomy was associated with significantly increased overall (aHR 1.17, 95% CI 1.03–1.33) and short-term (aHR 1.55, 95% CI 1.27–1.88) risk of CVD among individuals with BMI < 25 kg/m^2^, while it was not associated with increased CVD risk among individuals with BMI ≥ 25 kg/m^2^.

**Table 3:** Sensitivity analysis on the association of cholecystectomy with risk of cardiovascular disease among participants with fewer or more comorbidities.

	No cholecystectomy	Cholecystectomy	*P* value
Charlson comorbidity index < 2			
Cardiovascular disease			
Events	1069	73	
Person-years	93,595	5686	
aHR (95% CI)^a^	1.00 (reference)	1.03 (0.81–1.31)	0.831
Short-term (< 2 years) risk			
Cardiovascular disease			
Events	287	22	
Person-years	34,400	2117	
aHR (95% CI)^a^	1.00 (reference)	1.19 (0.76–1.86)	0.451
Long-term (≥ 2 years) risk			
Cardiovascular disease			
Events	782	51	
Person-years	93,315	5673	
aHR (95% CI)^a^	1.00 (reference)	0.97 (0.73–1.29)	0.838
Charlson comorbidity index ≥ 2			
Cardiovascular disease			
Events	2418	387	
Person-years	121,890	16,483	
aHR (95% CI)^a^	**1.00 (reference)**	**1.16 (1.04–1.29)**	**0.007**
Short-term (< 2 years) risk			
Cardiovascular disease			
Events	883	168	
Person-years	51,444	6748	
aHR (95% CI)^a^	**1.00 (reference)**	**1.47 (1.24–1.73)**	**< 0.0001**
Long-term (≥ 2 years) risk			
Cardiovascular disease			
Events	1535	219	
Person-years	121,028	16,354	
aHR (95% CI)^a^	1.00 (reference)	0.99 (0.86–1.15)	0.939

^a^Hazard ratios calculated by Cox proportional hazards regression analysis after adjustments for age, household income, alcohol consumption, body mass index, systolic blood pressure, fasting serum glucose, total cholesterol, Charlson comorbidity index.

Significant values are in bold.

**Table 4:** Sensitivity analysis on the association of cholecystectomy with risk of cardiovascular disease among women or men.

	No cholecystectomy	Cholecystectomy	*p* value
Women			
Cardiovascular disease			
Events	1291	159	
Person-years	94,133	9776	
aHR (95% CI)^a^	1.00 (reference)	1.03 (0.87–1.21)	0.755
Short-term (< 2 years) risk			
Cardiovascular disease			
Events	425	63	
Person-years	37,188	3842	
aHR (95% CI)^a^	1.00 (reference)	1.27 (0.97–1.66)	0.081
Long-term (≥ 2 years) risk			
Cardiovascular disease			
Events	866	96	
Person-years	93,712	9730	
aHR (95% CI)^a^	1.00 (reference)	0.91 (0.74–1.13)	0.413
Men			
Cardiovascular disease			
Events	2196	301	
Person-years	121,352	12,393	
aHR (95% CI)^a^	1.00 (reference)	1.12 (0.99–1.26)	0.084
Short-term (< 2 years) risk			
Cardiovascular disease			
Events	745	127	
Person-years	48,656	5023	
aHR (95% CI)^a^	**1.00 (reference)**	**1.39 (1.15–1.69)**	**0.001**
Long-term (≥ 2 years) risk			
Cardiovascular disease			
Events	1451	174	
Person-years	120,631	12,296	
aHR (95% CI)^a^	1.00 (reference)	0.97 (0.83–1.14)	0.733

^a^Hazard ratios calculated by Cox proportional hazards regression analysis after adjustments for age, household income, alcohol consumption, body mass index, systolic blood pressure, fasting serum glucose, total cholesterol, Charlson comorbidity index.

Significant values are in bold.

### Cholecystectomy and metabolic indices

Table 5 shows the changes in the cholecystectomy and control subjects’ metabolic indices such as total cholesterol, fasting serum glucose, systolic blood pressure, and body mass index during the same follow-up period.

**Table 5:** Differences in metabolic risk factors between cholecystectomy and no cholecystectomy groups.

	No cholecystectomy	Cholecystectomy	*p* value
Systolic blood pressure, mmHg			
Mean (SD)	**0.05 (17.36)**	− **1.41 (16.34)**	**< 0.0001**
aMean (95% CI)^a^	**0.24 (**− **0.8** to **0.57)**	− **1.31 (**− **1.87** to − **0.75)**	**< 0.0001**
aMean (95% CI)^b^	**3.20 (0.20** to **6.20)**	**1.51 (**− **1.50** to **4.51)**	**< 0.0001**
Total cholesterol, mg/dL			
Mean (SD)	− **1.31 (34.93)**	− **6.37 (36.12)**	**< 0.0001**
aMean (95% CI)^a^	− **1.07 (**− **1.74** to − **0.41)**	− **6.32 (**− **7.47** to − **5.17)**	**< 0.0001**
aMean (95% CI)^b^	− **8.91 (**− **15.09** to − **2.73))**	− **14.14 (**− **20.33** to − **7.95)**	**< 0.0001**
Fasting serum glucose, mg/dL			
Mean (SD)	0.08 (34.37)	0.30 (31.25)	0.720
aMean (95% CI)^a^	0.20 (− 0.45 to 0.84)	0.38 (− 0.72 to 1.49)	0.775
aMean (95% CI)^b^	2.48 (− 3.46 to 8.42)	2.69 (− 3.26 to 8.64)	0.755
Body mass index, kg/m^2^			
Mean (SD)	− **0.06 (1.35)**	− **0.18 (1.37)**	**< 0.0001**
aMean (95% CI)^a^	− **0.06 (**− **0.08** to − **0.03)**	− **0.18 (**− **0.23** to − **0.14)**	**< 0.0001**
aMean (95% CI)^b^	**0.00 (**− **0.24** to **0.23)**	− **0.13 (**− **0.37** to **0.11)**	**< 0.0001**

aMean calculated using linear regression.

^a^Adjusted for age and sex.

^b^Adjusted for age, sex, household income, smoking, alcohol consumption, exercise, and Charlson comorbidity index.

*SD* standard deviation.

Significant values are in bold.

Compared to the value of systolic blood pressure in patients who underwent cholecystectomy (aMean 1.51, 95% CI − 1.50 to 4.51), the value of systolic blood pressure in patients who did not undergo cholecystectomy tended to increase further to (aMean 3.20, 95% CI 0.20 to 6.20). The analysis results of total cholesterol showed a greater decrease in cholecystectomy patients to (aMean − 14.14, 95% CI − 20.33 to 7.95) than that in the general public (aMean − 8.91, 95% CI − 15.09 to 2.73). The BMI value showed a greater decrease in cholecystectomy patients (aMean − 0.13, 95% CI − 0.37 to 0.11) than in the no cholecystectomy group (aMean 0.00, 95% CI − 0.24 to 0.23).

## Discussion

Our large-scale, population-based retrospective cohort study showed that cholecystectomy was associated with a higher risk of developing cardiovascular disease, especially within two years of the surgery. This increased risk was more pronounced among males, those with BMI < 25 kg/m^2^, and those with more medical comorbidities with Charlson comorbidity index 2 or higher. Such increased risk of CVD was no longer observed when patients were followed for more than 2 years after surgery. Of note, cholecystectomy was associated with changes in systolic blood pressure, total cholesterol, and BMI following surgery.

**Figure 2:**
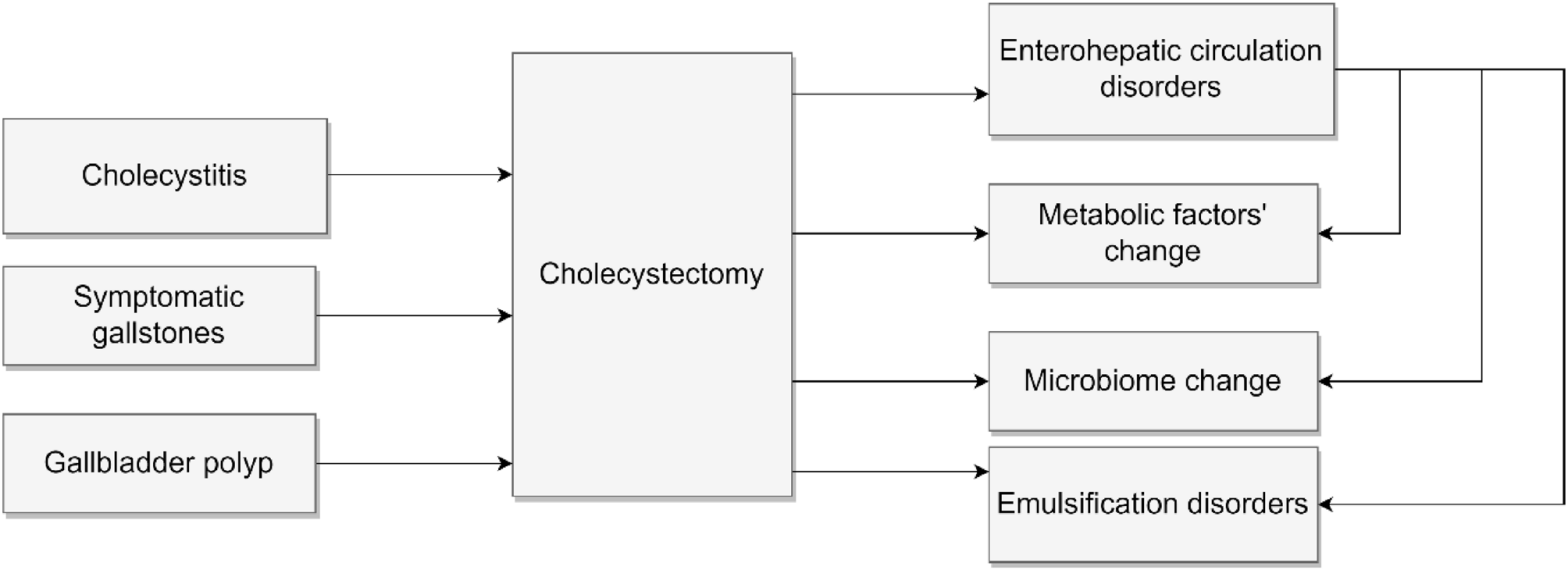
The mechanism of cholecystectomy and metabolism.

A previous study suggested that Total bile acids (TBA) are to be biomarkers of liver injury. So monitoring of IBA has been suggested for liver injury about differentiation of variety^33^. In Fig. 2, when cholecystectomy is performed, the first mechanism is to reduce the risk of cardiovascular disease by improving the intermediate metabolic profile due to inhibition of fat absorption or weight loss. Another mechanism is known to have a microbiome problem^3,34^. There is limitation to know whether mechanism is to reduce risk when the bile is gone, but there are probably primary biliary cirrhosis or mechanisms that cause liver damage or abdominal inflammation, but no other mechanisms have been identified. It can be shown that the mechanism of this study suggested that the no association in CVD risk after cholecystectomy is due to weight loss or improvement of intermediate metabolic profile.

In the previous study, the cholecystectomy group had higher risk of diabetes mellitus, hypertension and dyslipidemia than in the nonsurgical group^32,35^. The control group had a higher proportion of non-smokers than the surgical group^32^. This condition shows that the people who underwent cholecystectomy were those with high CVD risk.

From this point of view, previous studies have studied cancer in digestive organs such as colon cancer with cholecystectomy, and studies on degenerative brain diseases such as the relationship between cholecystectomy and Parkinson's disease. Based on this background, this study designed a study on whether cholecystectomy affected cardiovascular disease. In order to overcome the limitation that the conclusion of many papers so far has not found an intermediate explanatory stage between cholecystectomy and the disease of the resulting variable, we analyzed how cholecystectomy primarily affected our body's health metabolic index in short-term and analyzed its effect.

When extracted as a cholecystectomy subject, subjects who underwent cholecystectomy in a relatively healthy state were extracted except for those with a history of cancer and cardiovascular disease related to liver and gallbladder, but subjects with cholecystectomy were already high and body mass index was basically vulnerable to cardiovascular disease. As a result, CVD showed a high risk of development in subjects with cholecystectomy during the follow-up period from immediately after cholecystectomy to 2015, especially in CVD and CHD during the short period from immediately after surgery to 2 years. In addition, the amount of change in the body's metabolic index over a short period of time was analyzed, and as a result, most of the metabolic indicators tended to improve.

The existing hypothesis suggested that cholesterol can rise in the body by affecting cholesterol metabolism due to any effect in the metabolism of bile after undergoing cholecystectomy, and related existing studies have been conducted. Compared to previous studies, the first advantage of this study was that it looked before and after, and the limitation of the existing one-point study was that patients undergoing cholecystectomy were basically patients with poor metabolic indicators as cholesterol levels were high. In this study, basically, subjects who underwent cholecystectomy had relatively poor metabolic indicators before surgery than those who did not. However, after undergoing surgery called cholecystectomy, cholesterol dropped. Previous studies have assumed that cholecystectomy with only these mechanisms can cause abnormalities in bile metabolism and raise cholesterol levels, but when looking at this study the baseline had higher cholecystectomy subjects, but cholesterol levels or various indicators fell after surgery. Therefore, several existing papers have suggested two mechanisms related to cholesterol metabolism after cholecystectomy, but this study suggests that the part related to the hypothesis of cholesterol drop is more likely. That’s the conclusion of this study. However, the results at one point in the previous study are probably related to the metabolic high-risk group, but this study has a great advantage in that it considers the before and after comparison to accurately see the effect of cholecystectomy. Therefore, from the results of the above study, the overall beneficial effect of cholecystectomy is estimated in terms of cholesterol metabolism.

Furthermore, the risk of cardiovascular disease has decreased after 2 years in patients who underwent cholecystectomy, suggesting that cholecystectomy-associated elevation of cardiovascular disease risk may be ameliorated 2 years after cholecystectomy. Although there were concerns that cholecystectomy might have harmful effects in the past in metabolic indicators, such as cholesterol^12^, the results of this study suggest that there are more beneficial results.

### Study limitation

The limitation of this study is that it is not a total inspection DB because it is a retrospective cohort study. Since the study was not conducted with the total number of people, there is a limit to the application of the research results to the entire population due to the lack of population. Second, in the operational definition, patients who have undergone cholecystectomy may have been affected by each disease because they have underlying diseases such as gallbladder cancer, biliary tract cancer, and liver cancer, and remove them during liver transplantation. In addition, the effect of cardiovascular disease and underlying diseases related to liver and gallbladder may reflect the above analysis results. Third, it was explained that it is a bridge between cholecystectomy and disease, but the above results cannot explain whether the primary result of cholecystectomy is a change in metabolic indicators or its causality. Last, In the long-term analysis, cholecystectomy patients in our study represent an otherwise healthy sub-population with at least 2-years of survivorship, which may introduce type II error. To minimize survivor bias, however, both the case and controls were given at least 2-years of survival time. Therefore, it should be noted that it simply means association, and future studies suggest that studies should be conducted to explain the intermediate stage of cholecystectomy and disease of outcome variables.

### Clinical competencies

Cholecystectomy is a surgical procedure to remove organs. There are a lot of worries because it affects the body permanently. Some of previous studies suggested negative relationship between cholecystectomy and cardiovascular disease, Parkinson’s disease, and digestive cancer. However, the above study can relieve the worries of the previous research results. It can be interpreted that the short-term effect of cardiovascular disease due to cholecystectomy was affected by the patient’s preoperative health condition. In patients 2 years after cholecystectomy, unlike concerns caused by existing studies, the effect was improved in metabolic health after surgery. And after 2 years, the association between cholecystectomy and cardiovascular disease decreased.

### Translational outlook

Patients who have undergone cholecystectomy due to relatively non-critical diseases do not increase the risk of cardiovascular disease from a long-term perspective because metabolic indicators become healthy in the short term due to surgery.

### Supplementary Information

Supplementary Table 1.

## Data Availability

The dataset generated in the NHIS repository (https://nhiss.nhis.or.kr/).

## Abbreviations

CVD: Cardiovascular disease
CHD: Coronary heart disease
BMI: Body mass index
SBP: Systolic blood pressure
NHIS: National Health Insurance Service
ICD-10: International Classification of Diseases, Tenth Revision
CCI: Charlson comorbidity index
IRB: Institutional Review Board
aHR: Adjusted hazard ratio
CI: Confidence interval
