# Supplementary material for: Associations of cholecystectomy with metabolic health changes and incident cardiovascular disease: a retrospective cohort study
Source: Sci Rep. 2024 Feb 8;14:3195. doi: 10.1038/s41598-024-53161-6 (PMC10850095; doi:10.1038/s41598-024-53161-6)
Supplement: Supplementary file 1 — Supplementary Table 1. [file 41598_2024_53161_MOESM1_ESM.docx]

**Supplementary Table 1.** Sensitivity analysis on the association of cholecystectomy with risk of cardiovascular disease among participants without obesity.

|  | **No Cholecystectomy** | **Cholecystectomy** | ***p* value** |
| --- | --- | --- | --- |
| **Body mass index< 25 kg/m^2^** |  |  |  |
| Cardiovascular disease |  |  |  |
| Events | 2,198 | 275 |  |
| Person-years | 143937 | 13068 |  |
| aHR (95% CI) ^a^ | **1.00 (reference)** | **1.17(1.03-1.33)** | **0.017** |
| **Short-term (< 2 years) risk** |  |  |  |
| Cardiovascular disease |  |  |  |
| Events | 760 | 123 |  |
| Person-years | 57344 | 5210 |  |
| aHR (95% CI) ^a^ | **1.00 (reference)** | **1.55(1.27-1.88)** | **<.0001** |
| **Long-term (≥ 2 years) risk** |  |  |  |
| Cardiovascular disease |  |  |  |
| Events | 1,438 | 152 |  |
| Person-years | 143182 | 12970 |  |
| aHR (95% CI) ^a^ | 1.00 (reference) | 0.98(0.82-1.16) | 0.783 |
| **Body mass index ≥ 25 kg/m^2^** |  |  |  |
| Cardiovascular disease |  |  |  |
| Events | 1,289 | 185 |  |
| Person-years | 71548 | 9101 |  |
| aHR (95% CI) ^a^ | 1.00 (reference) | 0.98(0.84-1.15) | 0.825 |
| **Short-term (< 2 years) risk** |  |  |  |
| Cardiovascular disease |  |  |  |
| Events | 410 | 67 |  |
| Person-years | 28500 | 3655 |  |
| aHR (95% CI) ^a^ | 1.00 (reference) | 1.08(0.83-1.40) | 0.588 |
| **Long-term (≥ 2 years) risk** |  |  |  |
| Cardiovascular disease |  |  |  |
| Events | 879 | 118 |  |
| Person-years | 71161 | 9057 |  |
| aHR (95% CI) ^a^ | 1.00 (reference) | 0.94(0.77-1.14) | 0.522 |

^a^Hazard ratios calculated by Cox proportional hazards regression analysis after adjustments for age, household income, alcohol consumption, body mass index, systolic blood pressure, fasting serum glucose, total cholesterol, Charlson comorbidity index.
